# Examination of fluctuations in atmospheric pressure related to migraine

**DOI:** 10.1186/s40064-015-1592-4

**Published:** 2015-12-18

**Authors:** Hirohisa Okuma, Yumiko Okuma, Yasuhisa Kitagawa

**Affiliations:** Department of Neurology, Tokai University School of Medicine, Isehara, Japan; Green Heights Clinic, Kawasaki, Japan; Department of Neurology, Tokai University Hachioji Hospital, Tokyo, Japan

## Abstract

**Background:**

Japan has four seasons and many chances of low atmospheric pressure or approaches of typhoon, therefore it has been empirically known that the fluctuation of weather induces migraine in people. Generally, its mechanism has been interpreted as follows: physical loading, attributed by atmospheric pressure to human bodies, compresses or dilates human blood vessels, which leads to abnormality in blood flow and induces migraine. We report our examination of the stage in which migraine tends to be induced focusing on the variation of atmospheric pressure.

**Findings:**

Subjects were 34 patients with migraine, who were treated in our hospital. The patients included 31 females and three males, whose mean age was 32 ± 6.7. 22 patients had migraine with aura and 12 patients had migraine without aura. All of patients with migraine maintained a headache diary to record atmospheric pressures when they developed a migraine. The standard atmospheric pressure was defined as 1013 hPa, and with this value as the criterion, we investigated slight fluctuations in the atmospheric pressure when they developed a migraine. It was found that the atmospheric pressure when the patients developed a migraine was within 1003–1007 hPa in the approach of low atmospheric pressure and that the patients developed a migraine when the atmospheric pressure decreased by 6–10 hPa, slightly less than the standard atmospheric pressure.

**Conclusion:**

Small decreases of 6–10 hPa relative to the standard atmospheric pressure of 1013 hPa induced migraine attacks most frequently in patients with migraine.

## Background

The triggers of migraine are multi-factorial (Levy et al. 2009; Kelman 2007) and include weather changes, oversleeping, sleep deprivation, premenstrual period, stressful life events, hot/cold weather, relaxation after stress, menstruation, high wind, intense emotions, hunger, bright sunlight, red wine consumption, and food additives such as artificial sweeteners (e.g., aspartame), tyramine, monosodium glutamate, and nitrates. Weather changes are empirically known to trigger migraine (Gomersall and Stuart 1973; Cull 1981; Prince et al. 2004; Lilleng and Bekkelund 2009), and it is estimated that weather changes are involved in approximately 20 % of migraine episodes.

It is generally accepted that migraines can be caused by blood flow changes resulting from blood vessel dilatation associated with the influence of the changes in the physical load that atmospheric pressure imposes on the body. This may be particularly relevant in Japan, which has four seasons and is often hit by typhoons and cyclones. In this study, we investigated the relationship between migraine attacks and barometric pressure changes. Specifically, we report the range of pressure changes associated with migraine and we discuss methods for preventing migraines that develop in response to variations in barometric pressure.

## Methods

This study included 34 patients with migraine who received medical care at our hospital. The study population consisted of 31 females and three males, with a mean (±SD) age of 32 (±6.7) years. Twenty-two patients had migraine with aura (MA), whereas 12 had migraine without aura (MOA). In addition, a group of 28 patients with tension-type headache (TTH) were included as controls (Headache Classification Committee of the International Headache Society 2013).

The incidence of migraine in the study population was investigated from July 28, 2014, to August 15, 2014. During this period, a tropical cyclone (Typhoon No. 11, Halong) formed, approached, and passed across the Japanese archipelago. The central atmospheric pressure of this tropical cyclone ranged from 920 to 1008 hPa during the period. The patients were instructed to carry with them a headache diary to record the time of migraine attack and to check weather data released by the Japan Meteorological Agency and record the atmospheric pressure at the time of migraine onset. Standard atmospheric pressure was defined as 1013 hPa, and deviations from this value at the time of onset were calculated. The relationship between the onset of migraine and barometric pressure levels resulting from the approach of the tropical cyclone was examined using the Mann–Whitney U test.

The subjects were asked to refrain as much as possible from consuming alcoholic beverages and foods containing known migraine triggers during the study period.

## Results

The proportions of patients who developed migraine in association with atmospheric pressure decrease were as follows: 72.7 % (16/22) of MA patients, 75.0 % (9/12) of MOA patients, and 21.4 % (6/28) of TTH patients. Thus, patients with migraine experienced headaches associated with atmospheric pressure decrease at a significantly greater rate than patients with TTH. In addition, most of these migraines developed during periods of quite small pressure decreases from the standard atmospheric pressure (Table 1). Patients developed migraine at a rate of 23.5 % when the atmospheric pressure ranged from 1005 to <1007 hPa, and at a rate of 26.5 % when the atmospheric pressure ranged from 1003 to <1005 hPa. These proportions were significantly larger than those in controls (both *p* values were <0.05). Migraine occurred most frequently when the atmospheric pressure decreased by 6–10 hPa relative to the standard pressure. There was no significant difference between MA and MOA patients in the incidence of migraine attacks during atmospheric pressure decrease.

**Table 1:** The relationship between the atmospheric pressure and migraine incidence

	MA (n = 22)	MOA (n = 12)	Total (%)	TTH (n = 28) (control group)	*p* value
Gender (female)	21	10		19	ns
Ave. age	34 ± 4.8	33 ± 2.8		39 ± 6.4	ns
Atmospheric pressure (hPa) when developed headache					
1011 to <1013	0	1	2.9	0	ns
1009 to <1011	1	1	5.9	0	ns
1007 to <1009	1	0	2.9	0	ns
1005 to <1007	5	3	23.5	1	*p* < 0.05
1003 to <1005	7	2	26.5	1	*p* < 0.05
1001 to <1003	1	1	5.9	1	ns
999 to <1001	1	0	2.9	0	ns
997 to <999	0	1	2.9	0	ns
968 to <997	0	0	0.0	0	ns

*MA* migraine with aura, *MOA* migraine without aura, *TTH* tension-type headache, *ns* not significant

Patients developed migraine at a rate of 23.5 % when the atmospheric pressure ranged from 1005 to <1007 hPa, and at a rate of 26.5 % when the atmospheric pressure ranged from 1003 to <1005 hPa. These proportions were significantly larger than those in case of controls (both *p* values were <0.05)

## Discussion

Mukamal et al. (2009) analyzed 7054 patients admitted to an emergency department between May 2000 and December 2007 with a primary discharge diagnosis of headaches including migraine. They reported that a temperature increase of 5 °C in the 24 h preceding hospital presentation increased the acute risk of headache by 7.5 %. The occurrence of cyclones 48–72 h before hospitalization also increased the risk of non-migraine headache. The impact of barometric pressure change was greater in patients with migraine than in patients with non-migraine headache.

Alstadhaug et al. (2005) reported that migraine attacks occurred more frequently in the light arctic summer season than in the dark winter season, particularly in patients with a lifetime history of MA. They also reported an incidence rate of 80 %, a value comparable to ours. A distinctive feature of their study was that they focused on light intensity, particularly in patients with MA. In contrast, we focused on the association between migraine and barometric pressure. We found that atmospheric pressure changes are significantly associated with migraine attack.

We found that migraine often developed shortly before the appearance of cyclones. Specifically, we found that the range from 1003 to <1007 hPa, i.e., 6–10 hPa below standard atmospheric pressure, was most likely to induce migraine. In the study by Mukamal et al. (2009), the mean atmospheric variation was 7.9 mmHg, which is consistent with our finding. Despite many investigations (Kugler and Laub 1978; Cull 1981; Di Lorenzo et al. 2008; Larmande et al. 1996), it remains unclear how weather change triggers migraine. Bolay and Rapoport (2011) noted that low atmospheric pressure is associated with warm weather, winds, clouds, dust, and precipitation, and suggested that low pressure alone does not trigger migraine. They also proposed that high altitude may complicate the association of low atmospheric pressure with the triggering of migraine. In addition, they suggested that cortical spreading depression (CSD), an intrinsic brain event, activates prevascular trigeminal nerve fibers and may induce migraine (Bolay et al. 2002). On the other hand, Pun (2012) proposed that altitude-triggered migraine and high-altitude headache are likely due to hypobaric hypoxia rather than low atmospheric pressure. Messlinger et al. (2010) used a climate-controlled room to evaluate the effect of low atmospheric pressure on rats, specifically investigating whether rapid reduction of the ambient pressure to a level comparable to that during a typhoon would induce neuronal activity in the trigeminal nucleus caudalis. Their results indicate that rapid changes in barometric pressure activate trigeminal afferents, followed by an increase in arterial blood pressure. They found that such alteration of barometric pressure did not trigger trigeminovascular neurons with receptive fields from dura mater or from both dura mater and cornea; even prior sensitization with a nitric oxide donor did not alter the response to low atmospheric pressure.

In our study, patients with MA had a higher incidence of migraine attacks than patients with MOA. This is consistent with the idea that small atmospheric changes trigger migraine via the following mechanism (Welch et al. 1993; Lauritzen 1994; Lance et al. 1983; Silberstein and Silberstein 1990): (1) first, a small decrease in barometric pressure causes dilatation of cerebral blood vessels, leading to serotonin release from platelets, (2) increased levels of blood serotonin induce vasoconstriction and the onset of aura, and (3) finally, the subsequent decrease of serotonin causes rapid dilatation of cerebral blood vessels, thereby triggering migraine. This is consistent with the common observation that the temporary decrease in cerebral blood flow during CSD is caused by constriction of both the superficial and parenchymal blood vessels, whereas the post-CSD rise in cerebral blood flow is primarily related to the dilatation of parenchymal blood vessels. Considering that migraine patients were more susceptible to small atmospheric changes than controls, it is plausible that the sympathetic nervous system of patients with migraine is more sensitive to barometric perturbations and the pain threshold may be lower than that of controls.

We suggest that patients with barometric pressure-associated migraine should take note of daily weather forecasts and ensure that they have medication on hand if weather changes that might induce a migraine attack are likely to be encountered.

## Conclusion

We investigated the influence of decreases of the standard atmospheric pressure (1013 hPa) on frequency of migraine attacks in patients with migraine, and found that decreases of 6–10 hPa are most frequently associated with migraine. We recommend that when cyclones are forecast, patients with migraine should ensure that they have medication on hand.

## References

[CR1] Alstadhaug KB, Salvesen R, Bekkelumd SI (2005). Seasonal variation in migraine. Cephalagia.

[CR2] Bolay H, Rapoport A (2011). Does low atmospheric pressure independently trigger migraine?. Headache.

[CR3] Bolay H, Reuter U, Dunn A, Huang Z, Boas D, Moskowitz MA (2002). Intrinsic brain activity triggers trigeminal meningeal afferents in a migraine model. Nat Med.

[CR4] Cull RE (1981). Barometric pressure and other factors in migraine. Headache.

[CR5] Di Lorenzo C, Ambrosini A, Coppola G, Pierelli F (2008). Heat stress disorders and headache: a case of new daily persistent headache secondary to heat stroke. J Neurol Neurosurg Psychiatry.

[CR6] Gomersall JD, Stuart A (1973). Variations in migraine attacks with changes in weather conditions. Int J Biometeorol.

[CR7] Headache Classification Committee of the International Headache Society (2013). The international classification of headache disorders, 3rd edition (beta version). Cephalagia.

[CR8] Kelman L (2007). The triggers or precipitation of the acute migraine attack. Cephalalgia.

[CR9] Kugler J, Laub M (1978). Headache determination by meteorotropic influences. Res Clin Stud Headache.

[CR10] Lance JW, Lambert GA, Goadsby PJ, Duckworth JW (1983). Brainstem influences on the cephalic circulation: experimental data from cat and monkey of relevance to the mechanism of migraine. Headache.

[CR11] Larmande P, Hubert B, Sorabella A, Montigny E, Belin C, Gourdon D (1996). Influence of changes in climate and the calendar on the onset of a migraine crisis. Rev Neurol.

[CR12] Lauritzen M (1994). Pathophysiology of the migraine aura. The spreading depression theory. Brain.

[CR13] Levy D, Strassman AM, Burstein R (2009). A critical view on the role of migraine triggers in the genesis of migraine pain. Headache.

[CR14] Lilleng H, Bekkelund S (2009). Seasonal variation of migraine in an arctic population. Headache.

[CR15] Messlinger K, Funakubo M, Sato J, Mizumura K (2010). Increase in neuronal activity in rat spinal trigeminal nucleus following changes in barometric pressure—relevance for weather-associated headaches?. Headache.

[CR16] Mukamal K, Wellenius GA, Suh HH, Mittleman MA (2009). Weather and air pollution as triggers of severe headaches. Neurology.

[CR17] Prince PB, Rapoport AM, Sheftell FD, Tepper SJ, Bigal ME (2004). The effect of weather on headache. Headache.

[CR18] Pun M (2012). Migraine at altitude is it due to hypoxia or hypobaria?. Headache.

[CR19] Silberstein SD, Silberstein MM (1990). New concepts in the pathogenesis of headache. Pain Manag.

[CR20] Welch KM, Barkley GL, Tepley N, Ramadan NM (1993). Central neurogenic mechanisms of migraine. Neurology.

